# Polyp size measurement during colonoscopy using a virtual scale: variability and systematic differences

**DOI:** 10.1055/a-2371-3693

**Published:** 2024-09-23

**Authors:** Querijn N. E. van Bokhorst, Britt B. S. L. Houwen, Yark Hazewinkel, Manon van der Vlugt, Hanneke Beaumont, Joep Grootjans, Arjan van Tilburg, Paul Fockens, Patrick M. M. Bossuyt, Evelien Dekker, on behalf of the SCALE EYE study group, Marlou P. M. Adriaanse, Barbara A. J. Bastiaansen, Yvette H. van Beurden, Maxime E. S. Bronzwaer, Brecht W. E. Hens, Lowiek M. Hubers, Gem M. Kramer, Selma J. Lekkerkerker, Berrie Meijer, Fraukje A. Ponds, Dewkoemar Ramsoekh

**Affiliations:** 1 Department of Gastroenterology and Hepatology, Amsterdam UMC, Location VUmc, Amsterdam, the Netherlands; 2 Amsterdam Gastroenterology Endocrinology Metabolism, Amsterdam, the Netherlands; 3 Cancer Center Amsterdam, Amsterdam, the Netherlands; 4 Department of Gastroenterology and Hepatology, Tergooi Medical Center, Hilversum, the Netherlands; 5 Department of Gastroenterology, Bergman Clinics, Amsterdam, the Netherlands; 6 Oncode Institute, Amsterdam, the Netherlands; 7 Department of Pathology, Reinier de Graaf Gasthuis, Delft, the Netherlands; 8 Department of Clinical Epidemiology, Biostatistics and Bioinformatics, Amsterdam UMC, Location AMC, Amsterdam, the Netherlands; 9 Department of Gastroenterology and Hepatology, Dijklander Ziekenhuis, Hoorn and Purmerend, the Netherlands

## Abstract

**Background** Accurate polyp size measurement is important for polyp risk stratification and decision-making regarding polypectomy and surveillance. Recently, a virtual scale (VS) function has been developed that allows polyp size measurement through projection of an adaptive VS onto colorectal polyps during real-time endoscopy. We aimed to evaluate the VS in terms of variability and systematic differences.

**Methods** We conducted a video-based study with 120 colorectal polyps, measured by eight dedicated colorectal gastroenterologists (experts) and nine gastroenterology residents following endoscopy training (trainees). Three endoscopic measurement methods were compared: (1) visual, (2) snare and (3) VS measurement. We evaluated the method-specific variance (as measure of variability) in polyp size measurements and systematic differences between these methods.

**Results** Variance in polyp size measurements was significantly lower for VS measurements compared to visual and snare measurements for both experts (0.52 vs. 1.59 and 1.96, p < 0.001) and trainees (0.59 vs. 2.21 and 2.53, p < 0.001). VS measurement resulted in a higher percentage of polyps assigned to the same size category by all endoscopists compared to visual and snare measurements (experts: 69 % vs. 55 % and 59 %; trainees: 67 % vs. 51 % and 47 %) and reduced the maximum difference between individual endoscopists regarding the percentage of polyps assigned to the ≥ 10 mm size category (experts: 1.7 % vs. 10.0 % and 5.0 %; trainees: 2.5 % vs. 6.7 % and 11.7 %). Systematic differences between methods were < 0.5 mm.

**Conclusions** Use of the VS leads to lower polyp size measurement variability and more uniform polyp sizing by individual endoscopists compared to visual and snare measurements.

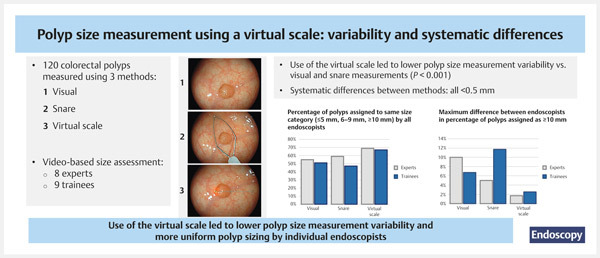

## Introduction

The size of colorectal polyps is associated with the risk that a polyp yields advanced histologic features 1 2 . Moreover, a polyp size ≥ 10 mm is associated with an increased risk of metachronous advanced neoplasia and colorectal cancer 3 . Therefore, accurate polyp size measurement, especially at the 10-mm threshold, is important for polyp risk stratification and decision making regarding colonoscopy surveillance intervals 4 5 . Polyp size also matters for deciding on optimal resection technique 6 7 and the safe implementation of the “leave-in-situ” and “resect-and-discard” optical diagnosis strategies 8 9 .

In daily practice, polyp size is mostly measured based on visual estimation by the endoscopist. However, visual size estimation is known to be inaccurate and prone to bias and interobserver variability 10 11 12 13 14 15 16 17 18 19 20 , reportedly leading to considerable missizing at relevant size thresholds and inappropriate surveillance recommendations for up to 35 % of polyps 10 12 19 . Using instruments of known size as a visual reference can improve polyp size measurement accuracy and reduce interobserver variability, but is known to be time consuming and costly 15 18 21 .

**Fig. 1:**
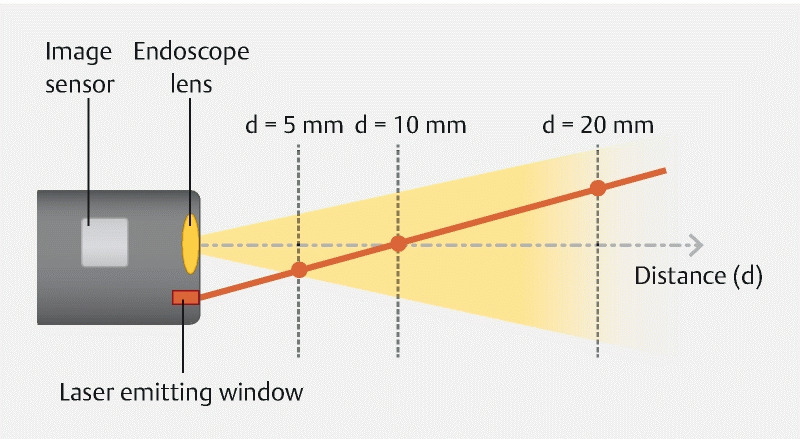
Schematic overview of the principle of distance estimation using the virtual scale endoscope. The reflection of the laser beam, as emitted from the endoscope, can be used to determine distance between the endoscope tip and the object (mucosal wall or polyp) the laser is positioned on based on the triangulation method. This information is used to continuously adapt the size of the virtual scale: the size of the virtual scale increases whenever the distance between the endoscope tip and the mucosal wall (or polyp) is shrinking, and decreases as the endoscope tips moves farther away. To enable real-time polyp size measurement, the virtual scale contains markings at 5, 10, and 20 mm.

Recently, a new virtual scale function (SCALE EYE; Fujifilm, Tokyo, Japan) was developed that allows polyp size measurement through projection of an adaptive virtual scale onto colorectal polyps during real-time endoscopy 22 . The size of the virtual scale is adapted based on the distance between the endoscope tip and the polyp the endoscopist aims to measure. This distance is calculated using an endoscope-integrated laser in combination with specific computer (image processing) software ( Fig. 1 ). The laser and the virtual scale can be simultaneously activated with a single push of a button on the handle of the endoscope.

Several (pre-)clinical studies have evaluated the performance of the virtual scale. These studies showed superior measurement accuracy of virtual scale polyp size measurements compared with both visual and instrument-aided polyp size measurements 17 18 19 20 21 22 23 . However, the clinical relevance of measurement accuracy as the primary outcome measure in evaluating polyp size measurement methods can be debated due to the absence of a robust reference standard for polyp size. To more thoroughly evaluate the clinical potential of the virtual scale, we aimed to evaluate the virtual scale in terms of variability and systematic differences. In addition, we evaluated feasibility of the virtual scale in terms of measurement success rate, measurement duration, and user-friendliness.

## Methods

### Setting and study design

The video-based study was conducted according to the principles of the Declaration of Helsinki and the Medical Research Involving Human Subjects Act (WMO) and was approved by the Institutional Review Board of the Academic Medical Center, Amsterdam (2022.084).

The size of consecutively detected polyps was measured in screening colonoscopies. All measurements were video recorded. Video recording extracts of these measurements were later presented to a group of both expert and trainee endoscopists. All endoscopists estimated the size of each polyp based on each of the three measurement methods: (1) visual estimation (without the aid of a tool), (2) 9-mm polypectomy snare as a visual reference, and (3) virtual scale as a visual reference. Polyps were also measured at histopathologic analysis.

### Colonoscopy procedures



**Video 1** Examples of endoscopic polyp size measurements using different measurement methods. In consecutive order: (1) visual measurement (without the aid of a tool); (2) measurement with the aid of the linear and circular virtual scale; (3) measurement with the aid of a polypectomy snare of known size (maximum width 9 mm).


Consecutive patients undergoing colonoscopy after a positive fecal immunochemical test within the context of the Dutch national colorectal cancer screening program at Bergman Clinics, Amsterdam were invited to participate in the study. All participants provided written informed consent. Patients were recruited between October 2022 and March 2023.

Colonoscopies were performed by four endoscopists (E.D., H.B., J.G., M.V.). All colonoscopies were performed using the EC-760S-A/L endoscope (Fujifilm) in combination with the EX-1 processor with EW10-VM01 software (Fujifilm), facilitating the use of the virtual scale. Endoscopists had measured at least 10 polyps using the virtual scale before the start of study inclusions.

**Fig. 2:**
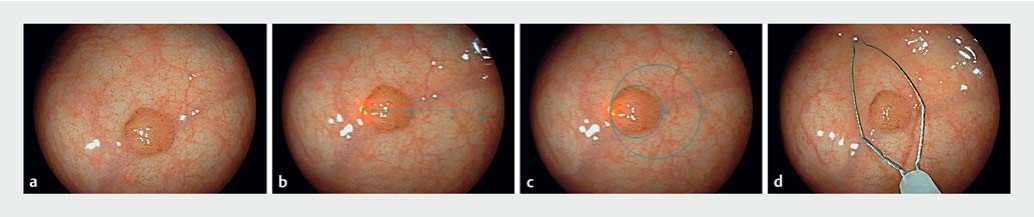
Endoscopic polyp size measurements methods. **a** Visual measurement (without the aid of a tool). **b** Measurement with the aid of the linear virtual scale. **c** Measurement with the aid of the circular virtual scale. **d** Measurement with the aid of a polypectomy snare of known size (maximum width of 9 mm).

As the upper limit of the virtual scale is 20 mm, only polyps of 20 mm or smaller, according to initial visual size estimation by the endoscopist, were deemed eligible for inclusion. Polyps were consecutively included, with the start of withdrawal within the cecum as the starting point. Eligible polyps were endoscopically measured using three methods and in similar order: (1) visual, (2) virtual scale, (3) snare ( Fig. 2 , Video 1 ). Measurements were performed using high definition white-light endoscopy.

Visual measurements were performed without the use of a tool as a visual reference. For virtual scale measurements, use of either the linear virtual scale, circular virtual scale, or both, was left to the discretion of the endoscopist. Duration of virtual scale measurements, defined as the time from activation of the virtual scale until the first image freeze with the virtual scale in a feasible position to estimate polyp size (i. e. on the left center edge of the polyp), was recorded. If no successful measurement could be performed within 180 seconds, the measurement was recorded as failed. For snare measurements, the Exacto Cold Snare (Steris, Mentor, Ohio, USA) with a maximum width of 9 mm (see **Fig. 1 s** , in the online-only Supplementary material) was used as a visual reference. The snare was positioned around the polyp while fully extended. If it was not possible to adequately position the snare around the polyp, the snare was positioned adjacent to the polyp. Snare measurements were recorded as failed if the snare could not be fully extended with both the polyp and complete snare clearly visible. All polyps were resected after snare measurement using the Exacto Cold Snare. Resection specimens were retrieved through suctioning and collected using a two-drawer polyp trap (ENDO-SAFIER Polyp Trap; Suzhou GZM Medical Co., Suzhou, China).

### Video-based assessment of polyp size

For each polyp, three 10–15-second video extracts were derived from the video recordings. Each extract included the measurement of a polyp using one of the three measurement methods. The video extracts were incorporated into an online survey environment ( **Fig. 2 s** ) and distributed over six surveys. Each survey contained 60 videos. All videos within each survey only showed polyp size measurements using one of the three measurement methods. The order of polyps within each survey was determined using a randomization tool 24 .

All video extracts were presented to eight dedicated colorectal gastroenterologists specialized in endoscopic diagnosis and treatment of early colorectal cancer and its precursor lesions (experts), as well as nine gastroenterology residents following endoscopy training with between 2 and 4 years of endoscopy experience (trainees). Before participation, all participants completed an e-learning program on use and interpretation of the virtual scale.

For each video, endoscopists were asked to report the size of the polyp displayed within the video. Size was reported in mm and at 1-mm increments. Playback and pausing of videos were allowed, as this mimics (re)positioning of the endoscope and image freezing during clinical procedures. To reduce the risk of recognition bias, intervals of at least 2 weeks were maintained between surveys containing video extracts of the same polyps. For each polyp, videos were presented to all endoscopists in a standardized order: (1) visual, (2) snare, (3) virtual scale. Endoscopists were also asked to grade the quality of each video for assessment of polyp size as “good,” “sufficient,” or “insufficient.” Endoscopists were blinded to the size as determined during clinical assessment and assessments by other endoscopists, and received no information on any results until completion of all surveys.

### Histopathologic polyp size measurement

Resection specimens of included polyps were collected in formalin and sent to the pathology laboratory. Resection specimens were measured by a dedicated gastrointestinal pathologist using a conventional ruler (macroscopic measurement) and during light microscope examination after embedment in paraffin and sectioning (microscopic measurement). In cases where the resection specimen was fragmented, this was recorded. Sizes were reported in mm at 1-mm increments.

### Statistical analysis

Continuous data are presented as mean with SD in cases of normally distributed data, and as median with interquartile range (IQR) in cases of non-normally distributed data. Categorical data are presented as count with percentages.

To estimate method-specific variance (as measure of variability), as well as systematic differences between methods, we used a mixed linear model (MLM). Our MLM included polyp size as the dependent variable, method-specific intercept as fixed effect, polyp-specific and endoscopist-specific intercepts as random effects, with a random slope to account for method-specific variance across different endoscopists. To test for a significant difference in variance between methods, we used the generalized likelihood ratio test statistic, comparing the model as described to a similar model presuming equal method-specific variances. In cases of significant differences in variance, MLM analyses were repeated on datasets comprising data of only two measurement methods. This way, any difference in variance between two specific methods could be evaluated in detail. To meet MLM assumptions of normality and homoscedasticity, log-transformed polyp size measurements were used for all hypothesis tests. To ease interpretation of the results, we report estimated variances and systematic differences without such transformation.

For subgroup analyses involving diminutive and nondiminutive polyps, mean polyp size according to snare measurements by expert endoscopists was used to distribute polyps over different size categories. For subgroup analyses with exclusion of polyps with videos of insufficient quality, polyps for which at least one video was rated as of insufficient quality by six or more (≥ 35 %) endoscopists were excluded.

We assessed uniformity of polyp size classification as the percentage of polyps assigned to the same size category (≤ 5 mm, 6–9 mm, ≥ 10 mm) by all endoscopists with each endoscopic measurement method. Differences in measured size between the various endoscopic measurement methods were illustrated using Bland–Altman plots 25 . Mean differences between endoscopic measurements and histopathologic measurements were calculated using the mean polyp size according to expert endoscopists for each endoscopic method. Polyps for which macroscopic and microscopic polyp size were not both available, as well as polyps that were resected in piecemeal or for which the histopathologic specimen was fragmented, were excluded from these analyses.

All analyses were performed using R version 4.2.1 (R Foundation for Statistical Computing, Vienna, Austria). Two-sided *P* values of < 0.05 were considered statistically significant.

### Sample size calculation

We based our sample size calculation on measurements by expert endoscopists, anticipating a 20 % or larger difference in variance between measurement methods. To reach the desired statistical power of 80 %, sample size calculation using a two-sided F-test for difference in variance with an alpha of 0.05 showed that inclusion of a total of 947 polyp measurements per method was required. When involving eight expert endoscopists, this implied inclusion of at least 119 unique polyps. To facilitate a balanced distribution of polyps over the six study surveys, we decided to extent the sample size to 120 polyps.

## Results

**Fig. 3:**
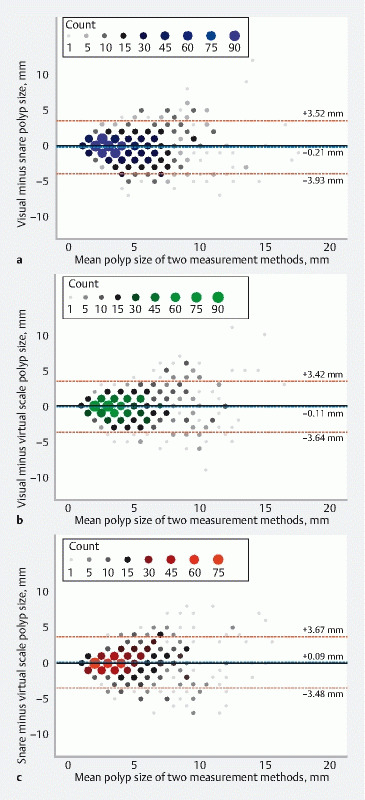
Bland–Altman plots illustrating the differences between polyp size measurements by expert endoscopists using different measurement methods. Within these plots, the polyp size according to two different measurement methods ( *x* axis) is plotted against the difference in polyp size according to these methods (y axis). Each plot comprises 960 observations, representing measurements of 120 polyps by eight different expert endoscopists. Count for each point within the plot is indicated by the legend on the top of each plot. Dotted blue lines represent mean differences. Dotted red lines represent upper and lower limits of the 95 %CIs. The plots represent the following methods ( *y* axis): **a** Visual and snare measurements. **b** Visual and virtual scale measurements. **c** Snare and virtual scale measurements.

A total of 120 polyps, detected during screening colonoscopy in 52 patients (median age 65 [IQR 60–71] years; male 62 %) were included ( **Table 1 s** , **Fig. 3 s** ). The success rate for virtual scale measurements was 95 %, which was comparable to the success rate for snare measurements (97 %). Median virtual scale measurement duration was 17 (IQR 8–33) seconds ( **Table 2 s** ). Median measurement duration was lower for the last 30 included polyps compared with the first 30 included polyps (12 [IQR 5–23] vs. 22 [IQR 14–44] seconds). Median measurement durations for flat and nonflat polyps were comparable (15 [IQR 6–30] vs. 18 [IQR 10–33] seconds). Five endoscopists who used the virtual scale in clinical practice graded virtual scale endoscope user-friendliness on average at 5 on a 10-point scale (with 1 representing the worst user-friendliness and 10 representing the best user-friendliness).

### Video-based assessment of polyp size

Eight experts (median endoscopy experience 13 [IQR 8–21] years) and nine trainees (median endoscopy experience 3 [IQR 2–4] years) completed all study surveys. For 97/120 polyps (81 %), all three measurement videos were assessed as being of sufficient to good quality. The percentage of videos with insufficient quality was comparable between the three measurement methods ( **Table 3 s** ).

### Differences in variability for endoscopic measurements methods

Method-specific variances are shown in Table 1 . Variance for virtual scale measurements was significantly lower than for visual and snare measurements for both experts (0.52 [95 %CI 0.47 to 0.57] vs. 1.96 [95 %CI 1.88 to 2.06] and 1.59 [95 %CI 1.50 to 1.67), *P* < 0.001) and trainees (0.59 [95 %CI 0.54 to 0.63] vs. 2.21 [95 %CI 2.12 to 2.30] and 2.53 [95 %CI 2.43 to 2.62], *P* < 0.001). One-to-one comparisons of method-specific variances showed statistically significant differences in all cases, with the exception of comparison of variance for visual vs. snare measurements for trainees.

**Table 1:** Variance for different endoscopic polyp size measurement methods as estimated using mixed linear model analyses.

Group	No. of measurements per method	Variance (95 %CI) 1		
		Visual	Snare	Virtual scale
Experts	960	1.96 (1.88 to 2.06)	1.59 (1.50 to 1.67)	0.52 (0.47 to 0.57)
Trainees	1080	2.21 (2.12 to 2.30)	2.53 (2.43 to 2.62)	0.59 (0.54 to 0.63)
All	2040	2.06 (1.99 to 2.12)	2.02 (1.96 to 2.09)	0.58 (0.52 to 0.64)

Difference in variance between methods (visual vs. snare, visual vs. virtual scale, and snare vs. virtual scale) was statistically significant in all cases ( *P* < 0.05), with the exception of the difference in variance between visual and snare measurements for trainees ( *P* = 0.21).

1 Variances are based on the squared deviations from the mean and are therefore reported in the square of the units of the original data (mm²). Standard deviations, expressed in the original units of the polyp size measurements (mm), can be calculated by taking the square root of the reported variances.

Subgroup analyses were conducted to evaluate variances within specific polyp subgroups. Polyps were subdivided based on polyp size (≤ 5 mm and > 5 mm polyps), morphology (flat and nonflat polyps), and the virtual scale used (linear or circular). These analyses showed similar results compared with the main analyses: variance for virtual scale measurements was lower than for visual and snare measurements in all cases, for both experts and trainees ( **Tables 4s–6 s** ). When excluding polyps with videos of insufficient quality, 97 polyps remained. Analyses involving this subgroup also revealed lower variance for virtual scale measurements compared with visual and snare measurements in both endoscopist groups ( **Table 7 s** ).

### Mean and systematic differences between measurement methods

Mean differences in polyp size between the three endoscopic measurement methods, based on assessments by expert endoscopists, are illustrated in Bland–Altman plots ( Fig. 3 ). These plots indicate that, on average, snare measurements resulted in the largest polyp size, respectively followed by virtual scale and visual measurements. Systematic differences between methods, as estimated using MLM analyses, are shown in Table 2 . For expert endoscopists, virtual scale measurements generally resulted in larger polyp size compared with visual measurements (+ 0.11 mm [95 %CI 0.00 to 0.23]) and smaller polyp size compared with snare measurements (–0.09 mm [95 %CI –0.21 to 0.02]).

**Table 2:** Systematic differences between different endoscopic polyp size measurement methods as estimated using mixed linear model analyses

Operator (no. of measurements per method)	Method one	Method two	Mean difference (95 %CI), mm 1
Experts (n = 960)			
	Visual	Snare	+ 0.21 (0.09 to 0.33)
	Visual	Virtual scale	+ 0.11 (0.00 to 0.23)
	Snare	Virtual scale	–0.09 (-0.21 to 0.02)
Trainees (n = 1080)			
	Visual	Snare	+ 0.36 (0.23 to 0.48)
	Visual	Virtual scale	+ 0.21 (0.08 to 0.33)
	Snare	Virtual scale	–0.15 (-0.27 to –0.03)
All (n = 2040)			
	Visual	Snare	+ 0.29 (0.20 to 0.37)
	Visual	Virtual scale	+ 0.16 (0.08 to 0.25)
	Snare	Virtual scale	–0.13 (-0.21 to –0.04)

1 Mean difference between polyp size measurements using method two compared with polyp size measurements using method one.

Mean differences between endoscopic and histopathologic measurement methods, based on the analysis of 71 nonfragmented polyps with both macroscopic and microscopic histopathologic measurement available, are shown in **Table 8 s** .

### Uniformity of polyp size classification for the different measurement methods

**Fig. 4:**
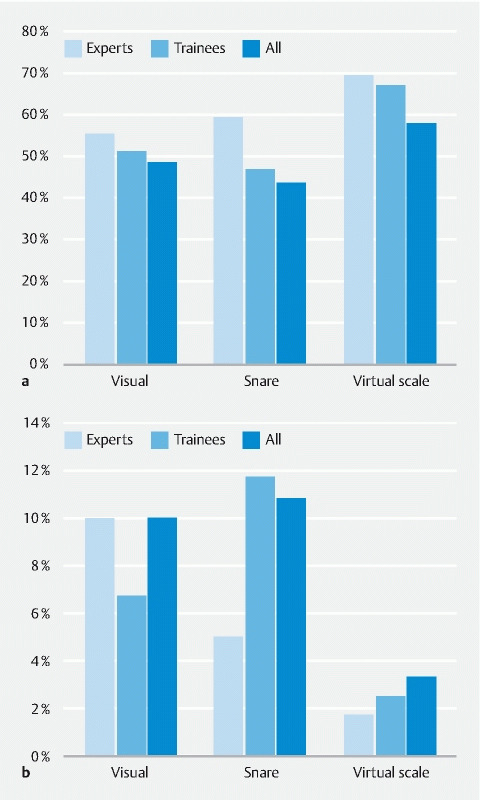
Bar plots. **a** The percentage of polyps assigned to the same category (≤ 5 mm, 6–9 mm, ≥ 10 mm) by all endoscopists using different polyp size measurements methods. **b** The maximum difference between individual endoscopists regarding the percentage of polyps assigned to the ≥ 10-mm size category.

Uniformity of polyp size classification was assessed through distribution of polyps over three size categories (≤ 5 mm, 6–9 mm, ≥ 10 mm) based on assessments by the individual endoscopists. The percentage of polyps assigned to the same size category by all endoscopists was higher for virtual scale measurements compared with visual and snare measurements for both experts (69 % vs. 55 % and 59 %), trainees (67 % vs. 51 % and 47 %), and all endoscopists (58 % vs. 48 % and 43 %) ( Fig. 4 **a** , **Table 9 s** ).

Use of the virtual scale resulted in more uniform decision making around the 10-mm threshold for both experts and trainees, as demonstrated by the maximum differences between individual endoscopists in the total number of polyps assigned to the ≥ 10-mm size category; this difference was lower for virtual scale measurements than for visual and snare measurements. For experts, based on analyses with 120 included polyps, a maximum difference regarding the number of polyps assigned to the ≥ 10-mm size category of 2 polyps (1.7 %) was found for virtual scale measurement, compared with 12 polyps (10.0 %) for visual measurement and 6 polyps (5.0 %) for snare measurement. For trainees, the maximum differences for virtual scale, visual, and snare measurement respectively concerned 3 (2.5 %), 8 (6.7 %), and 14 (11.7 %) polyps ( Fig. 4 **b** ). A more detailed overview of polyp distribution over the different size categories by individual endoscopists is shown in **Table 10 s** .

## Discussion

This video-based study showed that use of the virtual scale resulted in lower polyp size measurement variability compared with visual and snare measurements, for both endoscopy experts and trainees. This resulted in more uniform assignment of polyps to different size categories by individual endoscopists and a reduction in the differences in the number of polyps assigned to the ≥ 10-mm size category. Moreover, estimated systematic differences for virtual scale measurements compared with other methods were small (< 0.5 mm).

Most studies evaluating polyp size measurement methods use measurement accuracy as the primary outcome measure. However, the clinical value of measurement accuracy is compromised by the lack of a robust reference standard for polyp size. Frequently used reference standards are the measured size of (fresh) resection specimens 12 18 19 or size as endoscopically determined by in situ comparison of polyp size with the size of an instrument of known size 13 14 15 26 27 . A limitation of the use of resection specimen size as a reference standard is that resection specimens can become fragmented or lost. Moreover, the size of resection specimens might not represent real polyp size as these are prone to deformation due to vascular collapse, compression, cauterization, and suction 28 , as well as shrinkage due to formalin fixation 29 30 31 . Instrument-aided measurements can be erroneous due to problems with adequate positioning of instruments with the largest diameter in a perpendicular view toward the polyp. Instrument-aided measurements are also prone to bias owing to changed instrument proportions due to mechanic factors (e. g. compression, deformation) and distortion or warping of instruments on the endoscopy monitor due to the endoscope fisheye lens structure 32 . Our study illustrates that the latter may especially affect less experienced endoscopists, while variability for snare measurements exceeded variability for visual measurements for trainees. Given the drawbacks of comparing accuracies based on suboptimal reference standards, we chose to evaluate the different methods for polyp size measurement in terms of variability and systematic differences.

Lower measurement variability indicates that results of repeated measurements are concentrated closer around their mean. Use of measurement methods associated with lower measurement variability, such as the virtual scale in our study, will therefore result in a reduction of significant outliers and a lower rate of observer disagreement compared with measurement methods associated with higher measurement variability 33 . In our study, lower measurement variability for virtual scale measurements resulted in an increase in uniformity of polyp size category assignment up to 20 % compared with the other methods. The clinical relevance of the more uniform polyp sizing using the virtual scale is more specifically illustrated through the reduction in the percentual difference in the total number of polyps assigned to the ≥ 10-mm size category between individual endoscopists (i. e. experts: 10.0 % for visual to 1.7 % for virtual scale; trainees: 11.7 % for snare to 2.5 % for virtual scale). Sizing a polyp either under or over the 10-mm threshold may result in defining an adenoma as advanced (≥ 10 mm) or nonadvanced (< 10 mm), which in most guidelines implies a difference between either a 3– or 10-year surveillance interval 4 5 . As such, more consistent polyp sizing around the 10-mm threshold using the virtual scale could aid in preventing unnecessary (early) colonoscopies and erroneously delayed surveillance.

Despite benefits of the virtual scale in terms of measurement variability, this does not directly imply that virtual scale measurements also yield a high measurement accuracy. The small systematic differences (< 0.5 mm) as found in our study, in combination with results of preliminary preclinical studies evaluating performance of the virtual scale on artificial polyps (maximum measurement errors of 0.7 mm and relative accuracies of 82 %–84 %) 17 20 21 22 23 , do however support the idea that virtual scale measurements generally provide reliable estimates of real polyp size.

General benefits of the virtual scale include the fact that it is an intuitive push-a-button tool, which is easy to use and does not require additional (disposable) instruments. In addition, as shown in our study, virtual scale measurement is possible for the vast majority of polyps and can be performed in under a minute for 90 % of polyps. Although we did not compare virtual scale and snare measurement duration, the measurement duration when using the virtual scale is suggested to be comparable to other instrument-aided measurements in an ex vivo setting 21 . Nonetheless, there are also several factors compromising the usability of the virtual scale. First, maneuvering the virtual scale (endoscope) into a perpendicular position toward the polyp can be a tedious process and is not always possible, especially for polyps located in difficult positions (e. g. within folds). This is the main reason endoscopists graded user-friendliness on average at 5 on a 10-point scale. Moreover, the virtual scale only includes markings at 5, 10, and 20 mm, which hampers measurement of larger polyps. Finally, virtual scale measurements still require interpretation by physicians, thereby not completely ruling out bias due to interobserver variability.

In the future, the feasibility of the virtual scale as an alternative reference standard for clinical polyp size measurement should be evaluated considering its current limitations. This is particularly relevant given that artificial intelligence (AI) has already been proposed as an alternative for automated polyp size measurement. AI might facilitate polyp size measurements without bias due to human factors. However, the lack of robust datasets with ground truth information is currently still complicating development of AI-based polyp measurement systems 34 35 . In the context of lower measurement variability and proven measurement accuracy of the virtual scale on artificial polyps (of which exact size is known), the virtual scale might however open doors to development of certain datasets and further development of AI-based approaches.

To the best of our knowledge, this is the first study involving the virtual scale to evaluate multiple endoscopic polyp size measurement methods in terms of variability and systematic differences. Due to the prospective inclusion of consecutive polyps of different morphologic subtypes and sizes in a real-time clinical setting, our study provides realistic insights into the clinical potential of the virtual scale. In addition, both endoscopy experts and trainees participated in the study. To prevent recognition bias, we maintained intervals of at least 2 weeks between assessments of different measurements of the same polyps and applied video sequence randomization within surveys.

The generalizability of our study may however be compromised by the fact that the majority of polyps were ≤ 10 mm, and by the fact that participating trainee endoscopists had 2–4 years of endoscopy experience. Therefore, future studies involving more polyps over 10 mm in size and studies exploring the specific benefits of the virtual scale for novice endoscopists are still warranted. Moreover, while four endoscopists performed all study measurements, parameters such as measurement duration were largely dependent on the endoscopy skills of these endoscopists. Finally, polyp measurements were conducted based on video extracts with a maximum duration of 15 seconds. Although assessments based on videos are likely to be more accurate than assessments based on still images, the use of such short video extracts will always induce some bias. Nonetheless, analyses with exclusion of polyps with videos of insufficient quality showed results comparable to the results of the main analyses. This indicates that lower video quality was not the ground cause for observed differences in variability.

In conclusion, this study showed that use of the virtual scale led to lower polyp size measurement variability and more uniform polyp sizing by individual endoscopists compared with other measurement methods. Therefore, use of the virtual scale in daily clinical practice could reduce the risk of polyp under- or oversizing and polyp misclassification at relevant size thresholds. This could support better clinical decision making processes involving polyp size.

## Supplementary material

Supplementary material Zusatzmaterial
