# Supplementary material for: Polyp size measurement during colonoscopy using a virtual scale: variability and systematic differences
Source: Endoscopy. 2024 Sep 23;57(2):137–45. doi: 10.1055/a-2371-3693 (PMC11774581; doi:10.1055/a-2371-3693)
Supplement: Supplementary file 2 — Supplementary material [file 24034supmat_10-1055-a-2371-3693.pdf]

Supplementary material

Polyp size measurement during colonoscopy using a virtual scale: variability and systematic differences

Querijn N.E. van Bokhorst, Britt B.S.L. Houwen, Yark Hazewinkel, Manon van der Vlugt, Hanneke Beaumont, Joep Grootjans, Arjan van Tilburg, Paul Fockens, Patrick M.M. Bossuyt, Evelien Dekker on behalf of the SCALE EYE study group*

*Marlou P.M. Adriaanse, Barbara A.J. Bastiaansen, Yvette H. van Beurden, Maxime E.S. Bronzwaer, Brecht W.E. Hens, Lowiek M. Hubers, Gem M. Kramer, Selma J. Lekkerkerker, Berrie Meijer, Fraukje A. Ponds, Dewkoemar Ramsoekh

Supplementary material

Table 1s Polyp characteristics

Characteristic	Polyps, n (%)
Size*	
≤5 mm	83 (69)
6-9 mm	34 (28)
≥10 mm	3 (2.5)
Location†	
Right-sided colon	51 (43)
Transverse colon	23 (19)
Left-sided colon	46 (38)
Morphology according to Paris classification‡	
Ip	7 (5.8)
Ips	1 (0.8)
Is	52 (43)
0-IIa	56 (47)
0-IIb	4 (3.3)
Histology	
Adenoma	77 (64)
Sessile serrated lesion	6 (5.0)
Hyperplastic polyp	30 (25)
Other	7 (5.8)

*Based on mean polyp size according to snare measurements by expert endoscopists; †Right-sided colon consists of the cecum, ascending colon and hepatic flexure, the left-sided colon consists of the splenic flexure, descending colon, sigmoid and rectum; ‡Type 0-IIa and 0-IIb polyps were considered flat polyps and type Ip, Ips and Is polyps were considered non-flat polyps;

Table 2s Virtual scale measurement duration

Characteristic	Polyps, n (%)
Duration of virtual scale measurement in seconds, median (IQR)	17 (8–33)
Duration of virtual scale measurement in seconds	
<10	36 (30)
10–20	30 (25)
20–40	34 (28)
40–60	9 (7.5)
60–120	9 (7.5)
120–180	2 (1.7)

Supplementary material

Table 3s Video quality of video extracts according to assessment by different endoscopist groups

		Good, n (%)	Sufficient, n (%)	Insufficient, n (%)
**Experts**	Visual	507 (53)	364 (38)	89 (9.3)
	Snare	584 (61)	295 (31)	81 (8.4)
	Virtual scale	480 (50)	399 (42)	81 (8.4)
**Trainees**	Visual	669 (62)	332 (31)	79 (7.3)
	Snare	625 (58)	359 (33)	96 (8.9)
	Virtual scale	647 (60)	353 (33)	80 (7.4)
**All**	Visual	1176 (58)	696 (34)	168 (8.2)
	Snare	1209 (59)	654 (32)	177 (8.7)
	Virtual scale	1127 (55)	752 (37)	161 (7.9)

Notes: a total of 23 polyps had one or more video fragments of insufficient quality according to more than six endoscopists (≥35%). Video(s) of insufficient quality concerned the visual measurement video for seven polyps, the snare measurement video for six polyps, the virtual scale measurement video for six polyps, both the visual and virtual scale measurement video for two polyps, both the snare and virtual scale measurement video for one polyp, and all three measurement videos for one polyp.

Table 4s Variance for different endoscopic polyp size measurement method as estimated using mixed linear models – analyses for different polyp size groups

Size group	Endoscopist group	Measurements per method	Variance (95% CI)*		
			Visual	Snare	Virtual scale
**≤5 mm**	Experts	664	1.01 (0.94, 1.09)	0.75 (0.68, 0.81)	0.46 (0.41, 0.51)
	Trainees	747	1.01 (0.94, 1.09)	1.20 (1.12, 1.28)	0.59 (0.54, 0.65)
	All	1411	1.00 (0.95, 1.05)	0.98 (0.93, 1.03)	0.54 (0.49, 0.56)
**>5 mm**	Experts	296	4.25 (4.01, 4.48)	3.23 (3.03, 3.44)	0.78 (0.68, 0.88)
	Trainees	333	5.28 (5.03, 5.52)	5.43 (5.18, 5.68)	0.84 (0.61, 0.79)
	All	629	4.68 (4.51, 4.85)	4.29 (4.13, 4.46)	0.77 (0.70, 0.84)

CI, confidence interval; *Variances are based on the squared deviations from the mean and are therefore reported in the square of the units of the original data (mm^2). Standard deviations, expressed in the original units of the polyp size measurements (mm), can be calculated by taking the square root of the reported variances.

Notes: analyses including 83 diminutive (≤5 mm) polyps and 37 non-diminutive (>5 mm) polyps.

Supplementary material

Table 5s Variance for different endoscopic polyp size measurement method as estimated using mixed linear models – analyses for different polyp morphology groups

Morphology group	Endoscopist group	Measurements per method	Variance (95% CI)*		
			Visual	Snare	Virtual scale
**Flat**	Experts	480	2.07 (1.95, 2.20)	1.81 (1.69, 1.93)	0.20 (0.55, 0.69)
	Trainees	540	1.79 (1.68, 1.91)	2.76 (2.62, 2.90)	0.86 (0.79, 0.94)
	All	1020	1.89 (1.81, 1.98)	2.25 (2.16, 2.35)	0.76 (0.71, 0.81)
**Non-flat**	Experts	480	1.86 (1.73, 1.98)	1.35 (1.25, 1.46)	0.43 (0.37, 0.49)
	Trainees	540	2.64 (2.51, 2.78)	2.25 (2.12, 2.38)	0.38 (0.33, 0.43)
	All	1020	2.24 (2.15, 2.33)	1.77 (1.69, 1.85)	0.42 (0.38, 0.46)

CI, confidence interval; VS, virtual scale; *Variances are based on the squared deviations from the mean and are therefore reported in the square of the units of the original data (mm²). Standard deviations, expressed in the original units of the polyp size measurements (mm), can be calculated by taking the square root of the reported variances.

Notes: based on the Paris classification, type 0-IIa (n = 56) and type 0-IIb (n = 4) polyps were considered flat polyps and type Ip (n = 7), Ips (n = 1) and Is (n = 52) polyps were considered non-flat polyps.

Table 6s Variance for different endoscopic polyp size measurement method as estimated using mixed linear models – analyses for polyps measured using different variants of the virtual scale

VS group	Endoscopist group	Measurements per method	Variance (95% CI)*		
			Visual	Snare	Virtual scale
**Linear**	Experts	472	1.81 (1.68, 1.93)	1.48 (1.37, 1.59)	0.50 (0.43, 0.56)
	Trainees	531	1.98 (1.86, 2.10)	2.59 (2.45, 2.73)	0.59 (0.52, 0.66)
	All	1003	1.84 (1.76, 1.92)	1.92 (1.84, 2.01)	0.61 (0.56, 0.66)
**Circular**	Experts	346	2.50 (2.34, 2.66)	1.46 (1.34, 1.59)	0.59 (0.52, 0.67)
	Trainees	423	2.81 (2.65, 2.97)	2.46 (2.31, 2.61)	0.69 (0.61, 0.77)
	All	799	2.62 (2.51, 2.73)	2.00 (1.90, 2.10)	0.64 (0.58, 0.69)

CI, confidence interval; VS, virtual scale; *Variances are based on the squared deviations from the mean and are therefore reported in the square of the units of the original data (mm²). Standard deviations, expressed in the original units of the polyp size measurements (mm), can be calculated by taking the square root of the reported variances.

Notes: analyses including 57 polyps for which the measurement was performed using the linear VS and 47 for which the primary measurement was performed using the circular VS. Polyps that were measured using both the linear and circular VS (n = 16) were excluded from the analyses.

Supplementary material

Table 7s Variance for different endoscopic polyp size measurement methods as estimated by mixed linear model analyses – analyses with exclusion of polyps with at least one video of insufficient quality

Endoscopist group	Measurements per method	Variance (95% CI)*		
		Visual	Snare	Virtual scale
**Experts**	784	1.70 (1.61, 1.80)	1.47 (1.39, 1.56)	0.52 (0.47, 0.57)
**Trainees**	882	1.89 (1.80, 1.98)	2.38 (2.28, 2.49)	0.55 (0.50, 0.59)
**All**	1666	1.75 (1.69, 1.82)	1.88 (1.82, 1.95)	0.56 (0.53, 0.60)

CI, confidence interval; *Variances are based on the squared deviations from the mean and are therefore reported in the square of the units of the original data (mm^2). Standard deviations, expressed in the original units of the polyp size measurements (mm), can be calculated by taking the square root of the reported variances.

Notes: analyses including 97 polyps.

Table 8s. Mean differences between histopathological polyp size measurements and endoscopic polyp size measurements methods

Method one	Method two	Mean difference (95% CI)*
Macroscopic	Microscopic	-0.35 (-2.99, 2.28)
Macroscopic	Visual	-0.13 (-4.46, 4.19)
	Snare	-0.07 (-4.47, 4.34)
	Virtual scale	+0.01 (-3.77, 3.76)
Microscopic	Visual	+0.22 (-3.57, 4.01)
	Snare	+0.29 (-3.06, 3.64)
	Virtual scale	+0.35 (-2.70, 3.39)

CI, confidence interval; *Mean difference of method two compared to method one (in mm), based on mean polyp size of assessments by expert endoscopists.

Notes: macroscopic histopathological size was available for 115/120 (96%) polyps. Microscopic histopathological polyp size was available for 110/120 (92%) polyps. The resection specimen of 43/120 (41%) polyps was fragmented. Differences as shown within this table are based on analysis including 71 non-fragmented polyps with both macroscopic and microscopic measurements available.

Supplementary material

Table 9s Overview of polyp size category assignment according to different endoscopist groups and measurement methods

Group	Method	Size categories					
		Only <u><</u>5 mm, n (%)	<u><</u>5 and 6-9 mm, n (%)	Only 6-9 mm, n (%)	6-9 and <u>></u>10 mm, n (%)	Only <u>></u>10 mm, n (%)	All, n (%)
**Experts**	Visual	66 (55)	36 (30)	0 (0)	11 (9.2)	0 (0)	7 (5.8)
	Snare	61 (51)	37 (31)	8 (6.7)	9 (7.5)	2 (1.7)	3 (2.5)
	Virtual scale	71 (59)	32 (27)	7 (5.8)	5 (4.2)	5 (4.2)	0 (0.0)
**Trainees**	Visual	60 (50)	39 (33)	0 (0)	10 (8.3)	1 (0.8)	10 (8.3)
	Snare	53 (44)	44 (37)	2 (1.7)	11 (9.2)	1 (0.8)	9 (7.5)
	Virtual scale	64 (53)	36 (30)	10 (8.3)	3 (2.5)	6 (5.0)	1 (0.8)
**All**	Visual	58 (48)	38 (32)	0 (0.0)	8 (6.7)	0 (0.0)	16 (13)
	Snare	49 (41)	48 (40)	2 (1.6)	9 (7.5)	1 (0.8)	11 (9.2)
	Virtual scale	59 (49)	45 (38)	5 (4.2)	4 (3.3)	5 (4.2)	2 (1.7)

Notes: a polyp for which a size <u><</u>5 mm was reported by all endoscopists, is assigned to the '<u><</u>5 mm' group. If at least one of the measurements would have been 6-9 mm, the polyp would have been assigned to the '<u><</u>5 and 6-9 mm' group, etcetera.

 | © 2024. The author(s).

Supplementary material

Table 10s Number and percentage of polyps in each size category according to assessments of individual endoscopists using different polyp size measurement methods

	Visual			Snare			Virtual scale		
	≤5 mm, n (%)	6-9 mm, n (%)	≥10 mm, n (%)	≤5 mm, n (%)	6-9 mm, n (%)	≥10 mm, n (%)	≤5 mm, n (%)	6-9 mm, n (%)	≥10 mm, n (%)
	**Experts**								
Expert 1	83 (69)	27 (23)	10 (8.3)	83 (69)	34 (28)	3 (2.5)	88 (73)	26 (22)	6 (5.0)
Expert 2	91 (76)	26 (22)	3 (2.5)	84 (70)	32 (27)	4 (3.3)	93 (78)	22 (18)	5 (4.2)
Expert 3	86 (72)	25 (21)	9 (7.5)	83 (69)	33 (28)	4 (3.3)	88 (73)	25 (21)	7 (5.8)
Expert 4	84 (70)	26 (22)	10 (8.3)	79 (66)	37 (31)	4 (3.3)	88 (73)	25 (21)	7 (5.8)
Expert 5	101 (84)	14 (12)	5 (4.1)	83 (69)	31 (26)	6 (5.0)	85 (71)	28 (23)	7 (5.8)
Expert 6	89 (74)	26 (22)	5 (4.1)	79 (66)	33 (28)	8 (6.7)	81 (68)	32 (27)	7 (5.8)
Expert 7	101 (84)	15 (13)	4 (3.3)	85 (71)	31 (26)	4 (3.3)	96 (80)	18 (15)	6 (5.0)
Expert 8	81 (68)	24 (20)	15 (13)	84 (70)	27 (23)	9 (7.5)	90 (75)	23 (19)	7 (5.8)
**Maximum difference**	**20 (17)**	**13 (11)**	**12 (10)**	**6 (5.0)**	**10 (9.3)**	**6 (5.0)**	**15 (13)**	**14 (12)**	**2 (1.7)**
	**Trainees**								
Trainee 1	77 (64)	34 (28)	9 (7.5)	91 (76)	27 (23)	2 (1.7)	89 (74)	25 (21)	6 (5.0)
Trainee 2	84 (70)	28 (23)	8 (6.7)	70 (58)	41 (34)	9 (7.5)	79 (66)	33 (28)	8 (6.7)
Trainee 3	94 (78)	18 (15)	8 (6.7)	99 (83)	13 (11)	8 (6.7)	92 (77)	21 (18)	7 (5.8)
Trainee 4	81 (68)	29 (24)	10 (8.3)	69 (58)	42 (35)	9 (7.5)	86 (72)	27 (23)	7 (5.8)
Trainee 5	90 (75)	21 (18)	9 (7.5)	90 (75)	28 (23)	2 (1.7)	87 (73)	26 (22)	7 (5.8)
Trainee 6	85 (71)	22 (18)	13 (11)	72 (60)	32 (23)	16 (13)	79 (66)	32 (27)	9 (7.5)
Trainee 7	91 (76)	24 (20)	5 (4.2)	75 (63)	38 (32)	7 (5.8)	86 (72)	26 (22)	8 (6.7)
Trainee 8	91 (76)	21 (18)	8 (6.7)	87 (73)	20 (17)	13 (11)	92 (77)	20 (17)	8 (6.7)
Trainee 9	81 (68)	29 (24)	10 (8.3)	78 (65)	35 (29)	7 (5.8)	84 (70)	28 (23)	8 (6.7)
**Maximum difference**	**17 (14)**	**16 (13)**	**8 (6.7)**	**29 (24)**	**29 (24)**	**14 (12)**	**13 (11)**	**13 (11)**	**3 (2.5)**

Notes: for each size category (≤5 mm, 6-9 mm, ≥10 mm) and endoscopist category (expert or trainee) the highest number of polyps assigned to the size category is indicated in green and the lowest number is indicated in red.

Supplementary material

Fig. 1s Size of the Exacto Cold Snare (in mm).

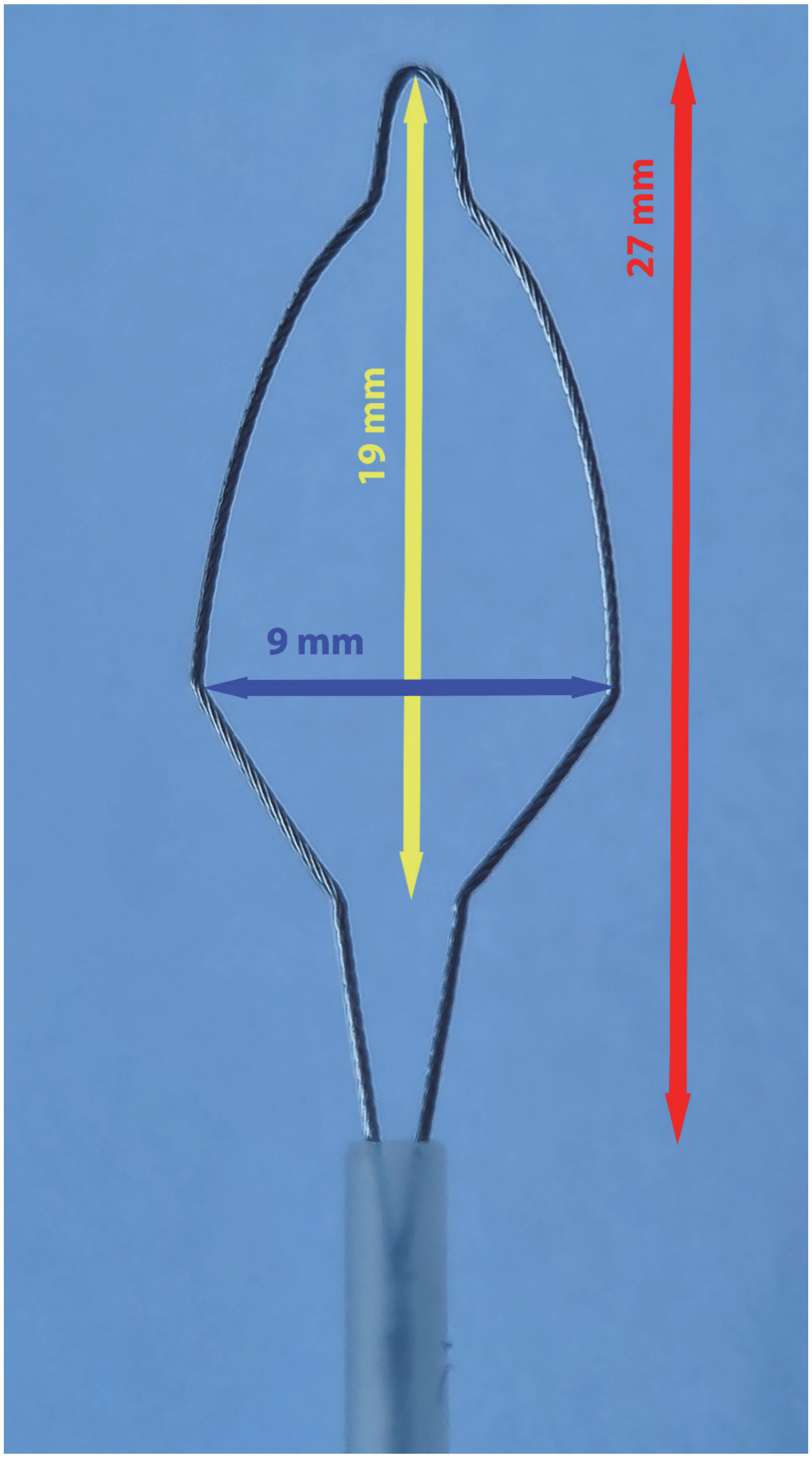

Supplementary material

Fig 2s Example of the survey environment: (A) video-based assessment of polyp size, (B) assessment of video quality for assessment of polyp size.

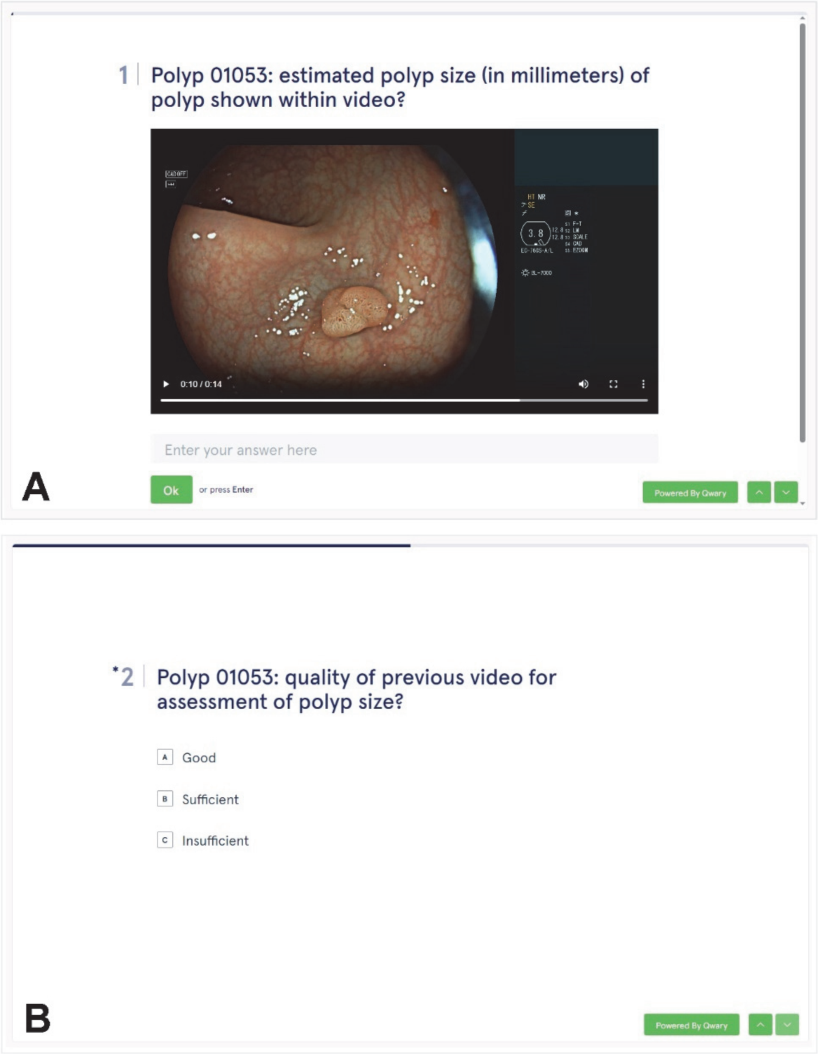

Supplementary material

Fig 3s Study flow chart.

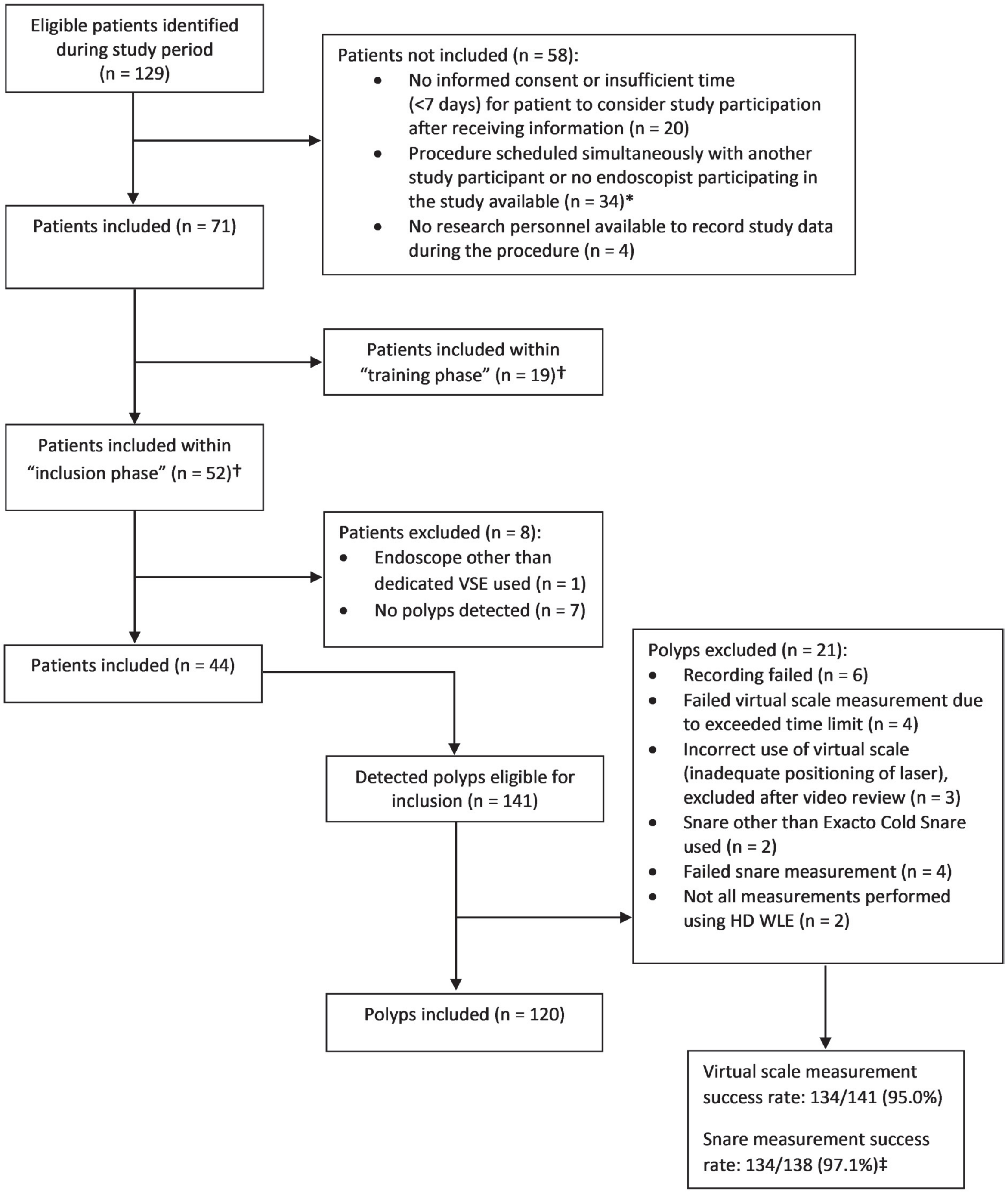

VSE, virtual scale endoscope; VS, virtual scale, HD WLE; high-definition white light endoscopy; *Only one endoscopy suite with the required study equipment was available; †All endoscopist had to perform at least 10 polyp size measurements by virtual scale ('training phase') before start of study inclusions ('inclusion phase'); ‡Measurements with a snare other than the dedicated study snare (Exacto Cold Snare) were excluded from the calculation.
